# Impact of NAD+ metabolism on ovarian aging

**DOI:** 10.1186/s12979-023-00398-w

**Published:** 2023-12-02

**Authors:** Jinghui Liang, Feiling Huang, Zhaoqi Song, Ruiyi Tang, Peng Zhang, Rong Chen

**Affiliations:** 1 Department of Obstetrics and Gynecology, Peking Union Medical College Hospital, Chinese Academy of Medical Sciences & Peking Union Medical College, National Clinical Research Center for Obstetric & Gynecologic Diseases, Beijing, 100730 China; 2 School of Medical Technology and Engineering, Fujian Medical University, Fuzhou, Fujian China; 3 Beijing Key Laboratory for Genetics of Birth Defects, Beijing Pediatric Research Institute, MOE Key Laboratory of Major Diseases in Children, Rare Disease Center, Beijing Children’s Hospital, Capital Medical University, National Center for Children’s Health, Beijing, 100045 China

**Keywords:** Ovary aging, Infertility, Metabolic homeostasis, NAD+ metabolism

## Abstract

Nicotinamide adenine dinucleotide (NAD+), a crucial coenzyme in cellular redox reactions, is closely associated with age-related functional degeneration and metabolic diseases. NAD exerts direct and indirect influences on many crucial cellular functions, including metabolic pathways, DNA repair, chromatin remodeling, cellular senescence, and immune cell functionality. These cellular processes and functions are essential for maintaining tissue and metabolic homeostasis, as well as healthy aging. Causality has been elucidated between a decline in NAD levels and multiple age-related diseases, which has been confirmed by various strategies aimed at increasing NAD levels in the preclinical setting. Ovarian aging is recognized as a natural process characterized by a decline in follicle number and function, resulting in decreased estrogen production and menopause. In this regard, it is necessary to address the many factors involved in this complicated procedure, which could improve fertility in women of advanced maternal age. Concerning the decrease in NAD+ levels as ovarian aging progresses, promising and exciting results are presented for strategies using NAD+ precursors to promote NAD+ biosynthesis, which could substantially improve oocyte quality and alleviate ovarian aging. Hence, to acquire further insights into NAD+ metabolism and biology, this review aims to probe the factors affecting ovarian aging, the characteristics of NAD+ precursors, and the current research status of NAD+ supplementation in ovarian aging. Specifically, by gaining a comprehensive understanding of these aspects, we are optimistic about the prominent progress that will be made in both research and therapy related to ovarian aging.

## Introduction

Ongoing advancements in NAD+ biology research continue to elucidate the mechanisms underlying age-related diseases. NAD+ , the reduced form of NAD, is an omnipresent coenzyme found in all human cells. It plays a crucial role in maintaining energy and redox homeostasis, regulating a vast network of systems across diverse cellular compartments and tissues [1–3]. In addition to its role in energy metabolism, NAD+ is recognized as a crucial signaling molecule and serves as the limiting substrate for numerous enzymes involved in DNA repair, epigenetic regulation, posttranslational modification, and metabolic adaptation [4]. The decline in NAD+ levels with aging has been thoroughly documented [5], and supplementation with NAD+ precursors have been shown to have the potential to elevate NAD+ levels, both in vitro and in vivo, serving as a promising strategy to combat age-related dysfunction and disease.

The association between NAD+ levels and health was initially established nearly a century ago by Elvehjem and his colleagues. In 1937, they discovered that pellagra resulted from a dietary insufficiency of niacin, resulting in reduced NAD+ levels [6]. Subsequent studies have demonstrated the correlation between low NAD+ levels and various disease conditions, such as metabolic disorders, neurodegenerative diseases, and aging [7–10]. Consequently, there is considerable interest in comprehending the influence of NAD+ metabolism on the initiation of diseases, particularly age-related conditions. In recent times, the restoration of NAD+ levels through the supplementation of NAD+ precursors has emerged as a promising therapeutic approach for age-related diseases [11–13], as evidenced by the beneficial effects observed in rodent models.

Ovarian aging, characterized by a decrease in both the quantity and quality of oocytes, along with an overall reduction in ovarian activity [14–16], poses a significant challenge to female reproductive health. Despite ongoing efforts, the molecular mechanisms responsible for ovarian aging and longevity remain largely unexplored, and the correlation between various factors and ovarian health necessitates further investigation. At birth, women have approximately 2 million oocytes, which diminish to a mere 1000 primordial follicles by menopause [17]. Delaying parenthood can lead to fertility issues for women of advanced maternal age, as their diminished ovarian reserve is linked to higher rates of aneuploidy and suboptimal outcomes in embryonic development and maturation following both natural conception and assisted reproductive technology [18, 19]. Recently, there has been a rapid increase in studies exploring various aspects of ovarian aging, including stress, genetics, diseases, dietary habits, and lifestyle. Obtaining a comprehensive understanding of these factors and their mechanisms is crucial for extending reproductive longevity and enhancing women’s health.

Strategies focused on decelerating ovarian aging and enhancing the quality and quantity of oocytes have made significant progress in recent decades [20–22]. Given that mitochondrial dysfunction and oxidative stress are pivotal factors in ovarian aging, the identification of drugs capable of mitigating ovarian disorders can play a crucial role in combating ovarian aging. These drugs can function as antioxidants or as molecules that modulate cellular signaling pathways to safeguard ovarian cells against oxidative stress. Examples of antioxidants include melatonin [23–26], coenzyme Q10 [27–30], folic acid [31], resveratrol [32, 33], and vitamins C and E [34, 35]. Although growth hormone (GH) is not classified as an antioxidant, it has the ability to impact the cellular-level oxidative stress signaling pathway [36, 37]. Employing small molecules or procedures involving mitochondrial transfer/replacement to enhance mitochondrial function has exhibited effectiveness in reducing oxidative damage to the ovaries [38].

In recent years, NAD has emerged as a promising regulator in mitigating age-related functional decline and diseases. In addition to improving mitochondrial function, this molecule enhances various other cellular processes and functions associated with antiaging effects. Encouragingly, certain studies have shown the potential of NAD precursors as a method of supplementing the body’s NAD levels. Moreover, the relationship between NAD+ levels and ovarian aging has gradually become clearer, with studies indicating that strategies to boost NAD+ can effectively alleviate ovarian aging, improve oocyte quality, and enhance fertility. Nevertheless, the specific mechanisms responsible for these effects remain unclear and necessitate further investigation and clarification [8, 39, 40]. This review offers a comprehensive overview of the current understanding of NAD+ biology and metabolism, the factors affecting ovarian aging, the NAD+ precursors, and the therapeutic potential of NAD+ boosting in countering ovarian aging.

## Factors affecting ovarian aging

### Mitochondrial dysfunction and oxidative stress

**Fig. 1:**
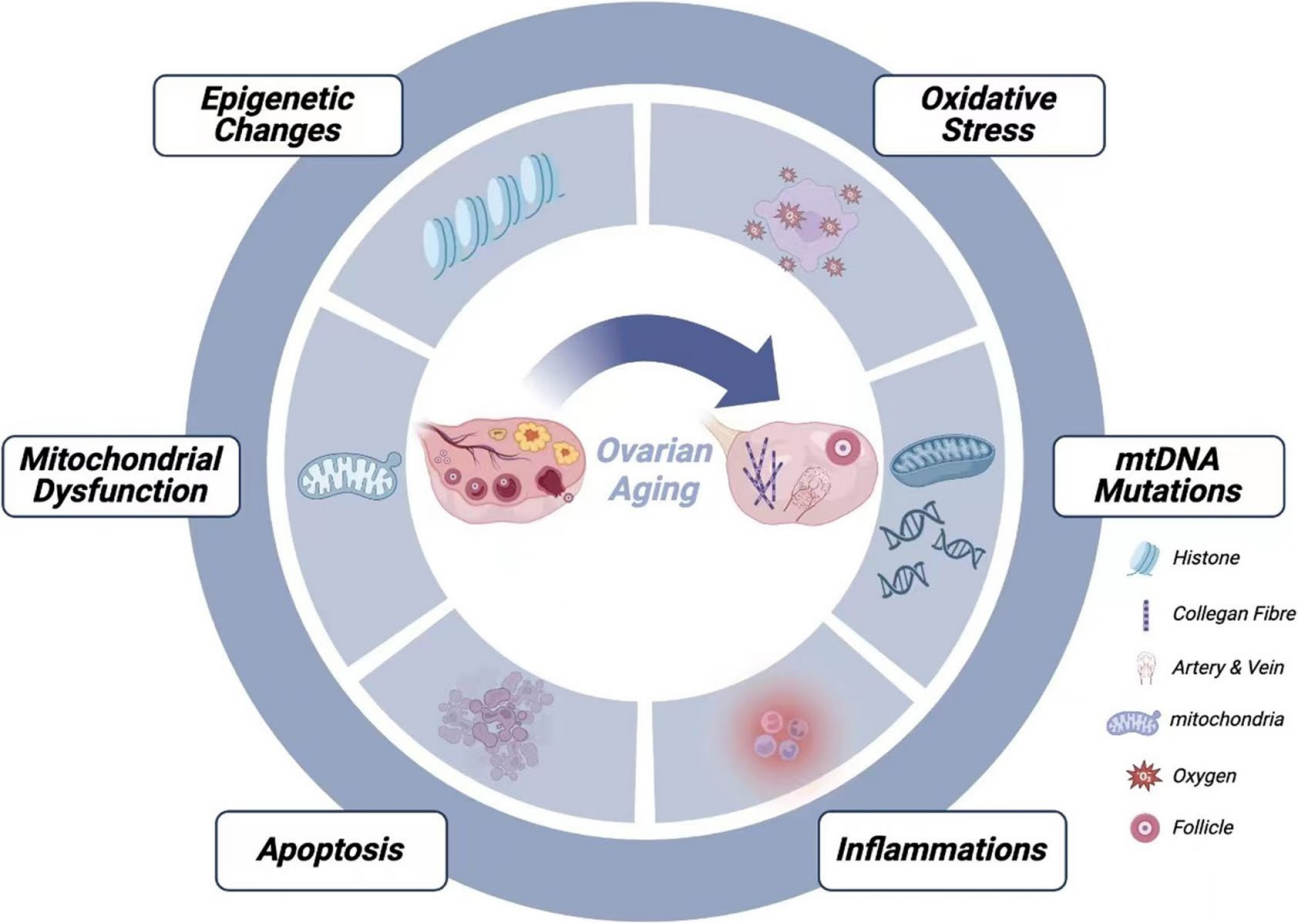
Factors affecting ovary aging. Many factors including mitochondrial dysfunction, apoptosis, inflammation, mtDNA mutations, oxidative stress and epigenetics changes have been shown linked to ovarian aging

The mitochondrion is a pivotal organelle in oocytes, playing a critical role in energy production and determining cell fate [41–44]. Being a semiautonomous structure with its DNA, the harmonious interaction between the nuclear and mitochondrial DNA is crucial for the proper functioning of the mitochondrion [45]. Impaired mitochondria, characterized by the accumulation of mtDNA mutations, reduced oxidative phosphorylation (OXPHOS) activity, increased oxidative damage, altered mitochondrial quality control, decreased biogenesis and clearance efficiency, and disrupted mitochondrial dynamics, have been linked to ovarian aging [46–49] (Fig. 1).

According to the free radical theory, oxidative stress, resulting from elevated levels of intracellular reactive oxygen species (ROS), is a crucial factor contributing to mammalian cell senescence, including female reproductive aging [50–53]. ROS encompass both free and nonfree radicals, primarily generated as byproducts during the metabolic processes of eukaryotes [54]. Moderate levels of ROS are known to be involved in cell signaling and can promote cell survival, proliferation, and differentiation [55, 56]. However, when ROS levels surpass a cell’s oxidation resistance and repair capabilities, they induce oxidative stress, causing direct oxidative damage to biological molecules in the cell environment and leading to aging and disease development [57, 58]. Due to the positive correlation between the levels of ROS in the ovary and a woman’s age [59–61], human oocytes, which remain dormant in the ovary for decades, are particularly vulnerable to oxidative stress. Endogenous antioxidants, such as superoxide dismutase and catalase, present in the ovarian environment are crucial for ROS clearance. However, their levels decrease with age, weakening the ovary’s ability to remove ROS [62–65]. A recent study using single-cell transcriptomics in nonhuman primates suggested that oxidative damage is a critical factor contributing to the age-related decline in ovarian function [66]. ROS accumulation in the ovary reduces communication between oocytes and GCs, triggering GC apoptosis [67–69], accelerating corpus luteum degeneration [70, 71], hindering oocyte maturation before ovulation [72, 73], and ultimately leading to ovarian aging. The well-established correlation between telomere length in CCs and oocyte and embryo quality [74] highlights that ovarian oxidative stress can cause telomere shortening [75, 76]. NAD plays a critical role in cellular redox reactions, and the decrease in NAD content is closely associated with mitochondrial dysfunction and the generation of oxidative damage.

### mtDNA disorders

Mitochondria play a critical role in synthesizing the energy-rich molecule adenosine triphosphate (ATP) through OXPHOS, providing energy to sustain cell activities [42, 77]. Electron leakage from the mitochondrial respiratory chain is a significant cause of intracellular ROS production. This susceptibility arises from the absence of protective histones or DNA-binding proteins in mtDNA, which is located within mitochondria, making it prone to ROS-induced damage [78–80]. ROS generation and mtDNA damage are closely intertwined, with the former often overlapping with the latter. As a consequence of ROS generation, mitochondrial fission, and mtDNA damage increase, primarily affecting the stromal side of the inner mitochondrial membrane [81, 82]. The gradual impairment in respiratory chain function resulting from mtDNA damage and mutations leads to an exponential increase in oxidative stress, especially with age. Female mice experience a shortened lifespan and exacerbated ovarian senescence due to the accumulation of mtDNA mutations in the germline [83, 84] (Fig. 1). Furthermore, younger women exhibit a higher mtDNA copy count per oocyte than that of elderly women, indicating reduced mtDNA in the ovary during aging [85]. Studies have demonstrated that autologous or allogeneic mitochondrial transplantation improves oocyte quality and in vitro fertilization outcomes in both human and other animal species [86, 87].

### Epigenetic changes

Epigenetic modifications are linked to the decline in oocyte quality with ovarian aging [88] (Fig. 1). The expression of DNA methyltransferases and histone acetyltransferases, which impact epigenetic modification in oocytes, changes with age [89, 90]. For instance, decreased expression levels of DNA methyltransferases in 35- to 40-week-old mouse oocytes and preimplantation embryos result in low DNA methylation levels [91]. Additionally, aging affects histone methylation in mouse germinal vesicle oocytes [92], while older women exhibit a lack of certain histone marks compared to those of younger women. The transcription of histone deacetylase is downregulated in aging mouse oocytes, while histones remain acetylated in 10-month-old female mouse oocytes [93]. This finding suggests that histone modification in aging oocytes before ovulation may be impacted, potentially leading to embryonic death during development. The mRNA expression profile of human second meiotic division oocytes is related to aging and has a greater negative impact on histone acetylation as the mother ages (van den [94]. There is growing evidence that microRNAs play a crucial role in regulating oocyte DNA methylation and follicle development across various species [95–98]. Disruptions in microRNA expression also contribute to the development of ovarian aging [99, 100]. Decreased cellular NAD levels lead to impaired function of NAD-dependent and NAD-consuming enzymes involved in DNA repair and genome integrity, potentially contributing to aging-related DNA mutations and epigenetic changes.

### Apoptosis

The ovary is a complex and heterogeneous organ composed of diverse cell types, with cumulus cells (CCs), playing a crucial role in the aging process. CCs, derived from granulosa cells (GCs), have been implicated in age-related elevations in oocyte apoptosis [101–103] (Fig. 1). The soluble molecules produced by CCs can adversely affect aging oocytes, leading to the acceleration of oocyte aging and impaired oocyte development and maturation potential [104, 105]. In vitro animal studies have demonstrated that coculture with CCs can improve oocyte maturation [106], while oocytes from older females have a marked decrease in the survival rate compared to that of oocytes from younger mice [107]. Studies in human patients have also revealed that GCs from young patients exhibit significantly lower levels of apoptosis than those from aged patients [108]. Moreover, animal experiments have shown that increased apoptosis levels in GCs result in a sharp decline in ovulation and fertility [109]. B-cell lymphoma-2 (BCL2), a key antiapoptotic factor, was found to be significantly upregulated in mature oocytes compared to immature oocytes, further reinforcing the role of CCs in accelerating the apoptosis in oocytes and aging of the ovary [110]. Recent research suggests that apoptosis and autophagy in aging cells contribute to the decline in NAD levels in the organism [111, 112].

### Inflammation

Recent research highlights inflammation as a hallmark of ovarian aging [113, 114] (Fig. 1). Inflammatory aging refers to a chronic, low-grade proinflammatory state that accompanies aging and impacts various aspects of ovarian aging, such as oocyte maturation [115, 116], ovulation [117, 118], implantation [119], and delivery [120]. Animal studies have revealed an increase in gene expression related to chronic inflammation with age [121–123]. During the aging process, researchers have observed an increase in the populations of CD4 + cells, B cells, and macrophages in the ovary, as well as serum concentrations and intraovarian mRNA levels of specific proinflammatory cytokines such as IL-1α/β, IL-6, and TNF-α and inflammasome genes such as ASC and NLRP3 [113]. Activation of the NLRP3 inflammasome is linked to age-related inflammation and dysfunction in various organs. Studies knocking out the NLRP3 and ASC genes showed a decrease in intraovarian proinflammatory cytokine expression and a significant increase in follicle numbers, indicating that inflammation contributes to the age-related decline in ovarian reserve and that anti-inflammation may prevent ovarian insufficiency [124–126]. Recent studies suggest that therapeutic approaches aimed at elevating cellular NAD levels during the aging process can effectively reduce inflammation and the burden of senescent cells [127].

### Telomeres length and telomerase activity

Telomeres are dynamic nucleoprotein-DNA structures located at the ends of eukaryotic chromosomes, crucial for maintaining genome integrity and chromosomal stability [128, 129]. Their length gradually shortens with each cell division. Telomerase is a reverse transcriptase enzyme that assists in elongating the highly repetitive DNA sequences of telomeres [130]. Studies have revealed that excessive telomere shortening contributes to cellular aging and is closely associated with reproductive lifespan and overall life expectancy [131, 132]. Research has found that in leukocytes of postmenopausal women, telomeres are shorter compared to age-matched women still experiencing menstruation. Additionally, women with longer telomeres tend to enter menopause at a later stage, indicating that telomere length serves as a significant marker of reproductive aging [133]. Investigations into human granulosa cells have linked telomere shortening and reduced or absent telomerase activity to latent ovarian insufficiency and primary ovarian insufficiency [134, 135].

Further research in assisted reproductive technology has shown that immature oocytes have significantly reduced telomere length compared to mature oocytes [136]. The length of telomeres in follicular cells exhibits a positive correlation with oocyte and embryo quality, and decreased telomere length may be associated with oocyte and early embryo aneuploidy [74, 137]. Oxidative stress is considered a major cause of telomere shortening [75]. With age, the levels of reactive oxygen species (ROS) increase in aging ovaries, making them more susceptible to telomeric oxidative damage and leading to a decline in oocyte developmental competence. The use of antioxidants can inhibit telomere shortening, fusion, DNA damage, and chromosomal instability in oocytes, thereby alleviating ROS-mediated damage and maintaining the quality of aging oocytes and follicles [138, 139].

## NAD+ biology and metabolism

### An introduction to NAD+ 

**Fig. 2:**
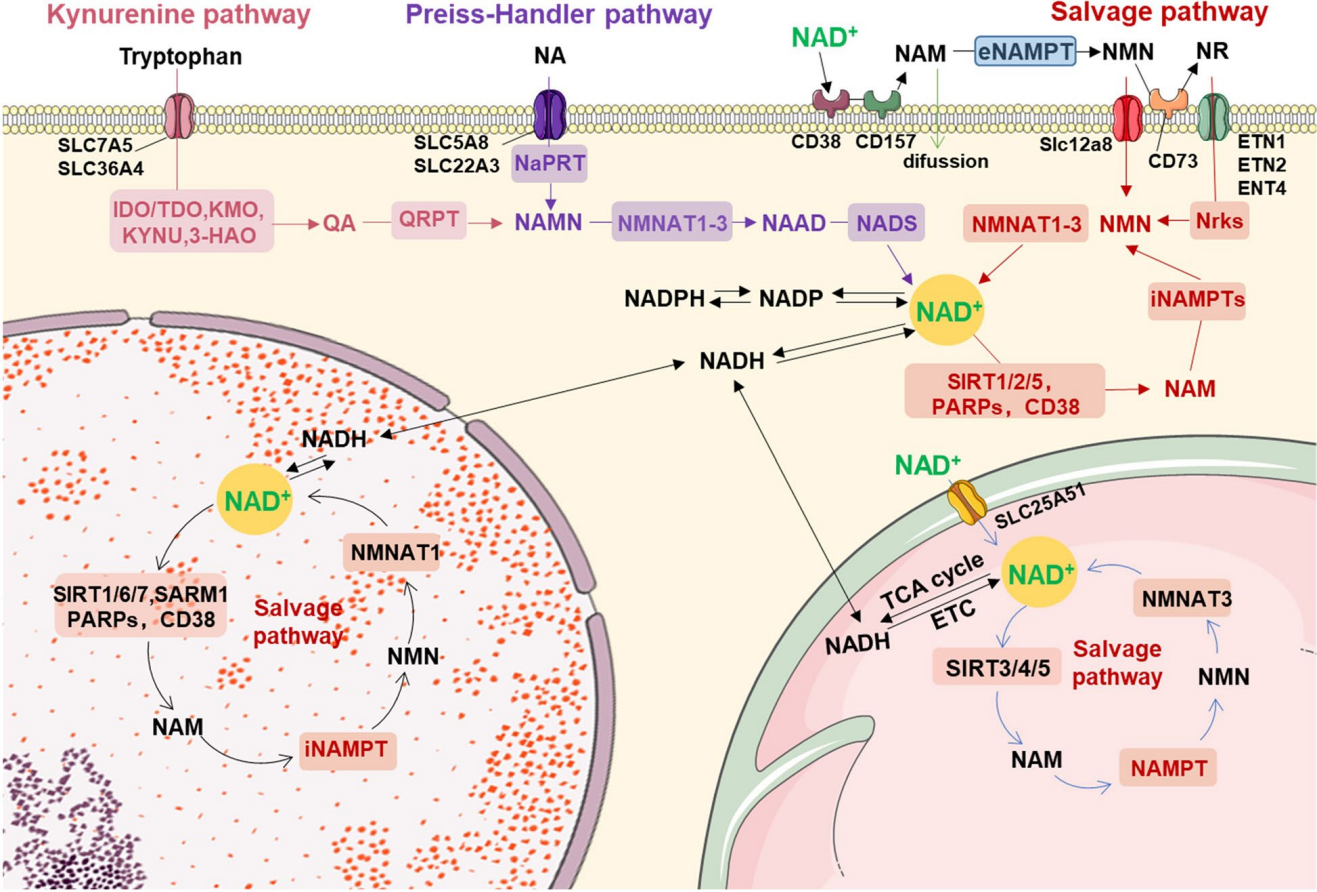
Overview of NAD+ metabolism pathway. NA: nicotinic acid, NAM: nicotinamide, QA: quinolinic acid, NMN: nicotinamide mononucleotide, NR: nicotinamide riboside, IDO, indoleamine 2,3-dioxygenase, TDO, tryptophan 2,3-dioxygenase, KMO, 3-hydroxykynurenine (3-HK) by kynurenine 3-monooxygenase, KYNU, tryptophan 2,3-dioxygenase, 3HAO. 3-hydroxyanthranilic acid oxygenase, ETC, NAMN: nicotinic acid mononucleotide, NAD: Nicotinamide adenine dinucleotide, NADH: reduced form of NAD, NADP: Nicotinamide adenine dinucleotide phosphate, NADPH: reduced form of NADP, NAAD: nicotinic acid adenine dinucleotide, NAMPT: nicotinamide phosphoribosyltransferase, NMNAT: nicotinamide mononucleotide adenylyltransferase, QPRT: quinolinic acid phosphoribosyltransferase, NaPRT: nicotinic acid phosphoribosyltransferase, Nrk: nicotinamide riboside kinase, NADS: NAD synthase, PARP: poly (ADP-ribose) polymerase, TCA, tricarboxylic acid, ETC, electron transport chain

NAD+ was first discovered in 1906 by Harden and Young as a low-molecular-weight substance that accelerates the fermentation of yeast extracts [140]. In 1930, its chemical composition was reported to be an adenine, a phosphate, and reducing sugar groups [141]. NAD+ was discovered in 1936 to possess the capability of hydride transfer between molecules, rendering it a crucial coenzyme in redox reactions and an indispensable constituent of the energy metabolism of all organisms [142]. NAD+ plays a regulatory role in the activity of dehydrogenases engaged in diverse catabolic pathways, including glycolysis, glutamine degradation, and fatty acid oxidation. Apart from its involvement in energy metabolism, NAD+ acts as a cofactor for nonredox NAD+ -dependent enzymes, including sirtuins, CD38, SARM1, poly(ADP-ribose) polymerases (PARPs), ADP-ribosyltransferases (ARTs), and RNA polymerases [143]. These enzymes are crucial in maintaining intracellular homeostasis [112, 144, 145]. Reactions utilizing NAD+ as a substrate or cofactor generate nicotinamide (NMA) as a byproduct, which is significant to numerous metabolic pathways and cellular processes. Extensive studies have revealed the intricate and dynamic nature of NAD+ metabolism, transport, and function, rendering it an area of continuous research [146–148]. NAD+ compartmentalization within cells is a complex phenomenon involving three primary NAD+ subcellular pools in the cytoplasm, mitochondria, and nucleus (Fig. 2). The exchangeability of NAD+ between the cytosolic and nuclear pools is well established, with these two pools consistently exhibiting comparable NAD+ concentrations [149–151]. However, the interchangeability of the mitochondrial NAD+ pool with the nucleocytosolic NAD+ pool remains a topic of debate in the scientific community. Evidence from yeast suggests the presence of mitochondrial NAD+ transporters [152], while studies in mammals indicate that mitochondria can take up NAD+ precursors and intact NAD(H) [153–155]. These findings imply that mitochondrial NAD+ pools can indeed exchange with other NAD+ pools. The proportion and regulation of these NAD+ pools can vary greatly depending on the organelle, tissue, cell type, and individual’s age, with enzymes related to NAD+ biosynthesis and degradation being highly compartmentalized and independently regulated [2, 149, 156].

### Synthesis of NAD+ 

In mammals, with neurons being the exception, NAD+ cannot be imported into cells [157]. Thus, NAD+ synthesis occurs through either the de novo pathway using tryptophan or the Preiss-Handler pathway involving vitamin B3 derivatives such as nicotinic acid (NA) (Fig. 2). The enzyme responsible for de novo NAD synthesis is predominantly expressed in the liver and kidney [158–161]. De novo synthesis is also called the kynurenine pathway, which begins with complex reactions converting tryptophan into quinolinic acid (QA) and then producing nicotinic acid mononucleotide (NAMN) from QA and 5-phosphoribosyl-1-pyrophosphate (PRPP) via the catalytic action of quinolinic acid phosphoribosyltransferase (QaPRT) [162]. NAMN can also be synthesized via the Preiss-Handler pathway, which utilizes vitamin B3 [163]. This molecule is converted into nicotinic acid adenine dinucleotide (NAAD) by nicotinamide mononucleotide adenylyl transferase (NMNAT). Subsequently, NAD+ synthase deamidates NAAD, leading to the formation of NAD+ [164].

To maintain intracellular NAD+ levels, the salvage pathway is the primary source of newly synthesized NAD+ , utilizing nicotinamide (NAM), nicotinamide riboside (NR), and nicotinamide mononucleotide (NMN). NAM can be obtained from food or produced by NAD+ -consuming enzymes [165]. It undergoes two reactions: first, NAM is catalytically converted into NMN via the catalytic action of nicotinamide phosphoribosyltransferase (NAMPT) and PRPP [166]. Then, NMN is converted into NAD+ by conjugating the adenylyl moiety of ATP, catalyzed by NMNAT [167]. NAMPT is widely distributed in all NAD+ -related cellular compartments and exhibits dynamic levels [168, 169]. This enables it to regulate the body’s response to nutritional status, stress, exercise, and circadian rhythm, all of which are relevant to the functions of NAD+ [170–172]. Therefore, NAM is widely regarded as a common NAD+ precursor in cells. Mammalian cells possess three NMNAT isoenzymes localized in different subcellular locations: NMNAT1 in the nucleus [173, 174], NMNAT2 in the cytoplasm and Golgi apparatus [167], and NMNAT3 in mitochondria [169, 175]. Conversely, NR is phosphorylated into NMN through nicotinamide riboside kinase (Nrk) phosphorylation before undergoing conversion into NAD+ by NAMPT [176].

### Degradation of NAD+ 

The sirtuin enzyme family is critical in regulating a wide range of biological processes, including metabolism, stress, circadian rhythm, and aging [177, 178]. These enzymes function as NAD+ -dependent deacetylases, utilizing NAD+ as a cosubstrate to remove acyl groups from their substrates and generating 2-O-acyl-ADP-ribose and NAM. In mammals, the sirtuin gene and protein family comprises seven members (SIRT1-SIRT7), each exhibiting distinct cellular localizations and functions [179, 180]. SIRT1 and SIRT6 reside in the nucleus, where they perform crucial roles in DNA repair and genome stability. In contrast, SIRT7 is specifically localized in the nucleolus. In mitochondria, SIRT3, SIRT4, and SIRT5 are responsible for regulating mitochondrial homeostasis. In the cytoplasm, SIRT1, SIRT2, and SIRT5 perform significant roles in circadian rhythm and gene expression. Based on their distinct Km values for NAD+ , sirtuins can be categorized into two groups: sirtuins with a Km value below the physiological range of NAD, such as SIRT2, SIRT4, SIRT5, and SIRT6, and sirtuins whose activity is highly dependent on the availability of NAD, including SIRT1 and SIRT3. Sirtuins are of great importance due to their ability to influence cell homeostasis through NAD+ levels, making them promising targets for antiaging therapy.

The PARP protein family serves as an essential consumer of NAD+ . This family consists of 17 members in humans and 16 members in mice, and it plays a pivotal role in DNA repair and genome integrity preservation. PARPs facilitate the cleavage of NAD+ to produce NAM and ADP-ribose. This ADP-ribose is subsequently attached to PARP and other receptor proteins, forming a polymer bond in a process referred to as poly(ADP-ribosyl) acylation (PAR acylation). PAR acylation is a dynamic, posttranscriptional modification that plays a crucial role in maintaining DNA repair and genome stability [181, 182]. Of the PARPs, PARP1, PARP2, and PARP3 are localized in the nucleus and consume a significant amount of NAD+ . As we age, both DNA damage and PARP activity increase. Among all PARPs, only PARP1, PARP2, and PARP3 are located within the nucleus. Of these, PARP1 and PARP2 are the primary consumers of NAD+ . Upon DNA damage, PARP1 is the sole contributor to approximately 90% of PARP activity [181]. Because of the fact that the Km value of PARPs is significantly lower than the physiological range of NAD, PARPs have a greater advantage in competing for the limited NAD resources than sirtuins [183, 184].

The cyclic ADP-ribose (cADPR) synthases constitute another group of NAD+ consumers, with CD38 and CD157 being the most prominent in cells. They possess both glycohydrolase and ADP-ribosyl cyclase activities, breaking down NAD+ into NAM and adenosine ADP-ribose and then producing cADPR [185]. Acting as an intercellular messenger, cADPR impacts calcium signal transduction, ROS production, and apoptosis [186]. In addition to NAD+ , CD38, and CD157 can also employ NMN and NR as alternative substrates, respectively [187–189]. Therefore, inhibitors of CD38 and CD157 hold the potential in restoring NAD+ levels in aging individuals and treating metabolic disorders and aging-related diseases [190]. The aging process causes an elevation in the levels of CD38 and CD157, leading to increased utilization of NAD+ [188, 191]. This phenomenon contributes to the decreased NAD+ levels observed in aged mice compared to young mice. Studies have demonstrated that CD38 deficiency in mice eliminates the reduction in NAD+ during aging [192], thereby indicating its potential role in aging-related diseases [112, 193].

SARM1, primarily expressed in neurons, is classified as an NAD+ glycohydrolase and cyclase, and its degradation of NAD+ is closely tied to axonal destruction [194, 195]. NAD+ is additionally involved in the formation of NAD+ RNA caps,however, its physiological relevance remains to be elucidated [196, 197].

## Function of NAD+ 

### Metabolism

NAD+ is an indispensable coenzyme intricately involved in cellular redox reactions, assuming an important role in cellular energy metabolism. It is engaged in diverse catabolic pathways, such as glycolysis, amino acid degradation, and fatty acid oxidation. Within these biochemical processes, NAD+ is reduced by accepting hydride ions, forming NADH. Subsequently, NADH transfers the acquired electrons to the electron transport chain, culminating in the production of ATP. Beyond its fundamental role in redox reactions, NAD+ exhibits versatility by undergoing phosphorylation to yield NADP. This phosphorylated form acts as a hydrogen acceptor, giving rise to NADPH, a pivotal participant in antioxidant defense and synthetic metabolic pathways.

Emerging evidence suggests that strategies aimed at modulating NAD+ degradation pathways or enhancing NAD+ levels may offer therapeutic benefits for metabolic disorders and aging [2]. Maintaining the balance of NAD+ is essential for the optimal functioning of diverse metabolic tissues [198]. Changes or disruptions in metabolic states, such as high-fat diets, postpartum weight loss, and circadian rhythm disturbances [154, 199–201], may lead to a decline in NAD+ levels, affecting NAD+ -dependent cellular processes. Conversely, increasing cellular NAD+ levels through exercise, caloric restriction, and healthy dietary interventions have been shown to reduce stress and promote metabolic normalization [202].

Knocking out PARP1 or CD38 or using PARP and CD38 inhibitors in mice leads to supraphysiological levels of NAD+ in vivo. This change enhances metabolic rates in mice during high-fat diet feeding and aging, and glucose metabolism remains relatively normal, demonstrating favorable effects in preventing obesity [188, 203, 204]. In mice subjected to a high-fat diet, reduced expression of NAMPT, a key enzyme in the NAD+ salvage pathway, leads to decreased activity in this pathway, which may be a potential mechanism underlying the decline in NAD+ levels during obesity. Additionally, in mice with adipocyte-specific NAMPT deficiency, NAD+ levels decrease, insulin resistance increases, and metabolic dysfunction worsens in adipose tissue [205], further supporting the significance of NAD + homeostasis in normal metabolic activities. The longevity protein SIRT2 has been found to promote lifespan in an NAD+ -dependent manner [177, 206]. Higher NAD+ levels also enhance the activity of nuclear SIRT1 and mitochondrial SIRT3, thereby regulating mitochondrial function and preventing diet-induced metabolic disorders [207, 208].

### Inflammation and immunity

The relationship between chronic inflammation, immune cells, and metabolic cells is complex [209]. Targeting macrophage immune metabolic pathways by modulating NAD+ biosynthesis or degradation is of significant importance in regulating the inflammatory state and alleviating diseases [210]. Recent studies have indicated that NAD+ is a crucial regulatory factor for macrophage function, and pro-inflammatory M1-like macrophages may serve as the primary source of pro-inflammatory cytokines in aging tissues. Enhanced CD38 expression in macrophages leads to increased NAD + consumption, resulting in pro-inflammatory (M1) macrophage polarization. Blocking NAMPT can impede glycolytic switching in M1 macrophages, limit pro-inflammatory responses in vitro, and reduce systemic inflammation in vivo. Conversely, increased NAMPT function leading to elevated NAD + levels promotes anti-inflammatory (M2) macrophage polarization [112].

During the aging process, elevated CD38 expression and increased NADase activity in the liver and adipose tissues contribute to declining NAD + levels and accumulation of pro-inflammatory M1-like macrophages [111, 211]. Impaired de novo NAD + synthesis in aging macrophages also affects their functionality during aging [212]. The enhanced expression of pro-inflammatory cytokines may drive a vicious cycle of inflammation, exacerbating tissue and DNA damage, and further activating major NAD + consumers such as CD38 and PARP, accelerating age-related physiological decline.

On the other hand, NAD + has been implicated in inducing cell death in specific T cell subsets [213], and it can also influence T cell polarization [214, 215], showing the dual role of NAD + in immune regulation. The precise role of NAD + in adaptive immune function remains unclear and requires further investigation.

### DNA repair, transcriptional regulation and epigenetics

As discussed in the section “Degradation of NAD + ” above, the PARP protein family, a crucial NAD + consumer, plays a central role in DNA repair and genome integrity. PARP accumulates at sites of single-strand breaks in cellular DNA and initiates the DNA repair process by utilizing NAD + for auto-ADP-ribosylation. Therefore, PARP is also considered a major consumer of NAD + during the aging process. Overactivation of PARP can be observed during aging or after DNA damage [216, 217], and this excessive activation may contribute to age-dependent NAD + depletion. Pharmacological inhibition or genetic deficiency of PARP1 prevents the decline in NAD + levels during aging and nutrient stress [217, 218]. NADP, acting as an endogenous inhibitor of PARP in mammalian cells, has been shown to be a negative regulator of PARylation and DNA damage repair in cancer cells [219]. Supplementing NAD + precursors can reduce DNA damage observed in hippocampal neurons of Alzheimer’s disease mouse models [220]. Apart from its role in DNA repair, PARP also functions as a chromatin modifier, a co-regulator of DNA-binding transcription factors, and a regulator of DNA methylation during the process of protein transcription [221–224]. Striking a balance between promoting and inhibiting PARP activity to achieve DNA repair and protein transcription regulation is crucial for inhibiting aging.

The sirtuin enzyme family, as another major NAD + consumer, is not only involved in DNA damage repair processes but also associated with epigenetic modifications related to aging. Sirtuins prevent DNA damage by inhibiting ROS production in mitochondria and activating ROS scavenging enzymes [225, 226]. They also promote DNA damage repair through mechanisms such as PARP activation [227], glutamine anaplerosis [228], and homologous recombination-mediated double-strand DNA break repair [229, 230]. The most notable role of sirtuins in epigenetics is their deacetylation function on histones. Deacetylation of histones H4K16, H3K9, and H3K56 by sirtuins contributes to lifespan extension [231–233]. Sirtuins can also activate histone methyltransferases and promote histone methylation processes [234].

### Cellular senescence

As NAD + levels decline, senescent cells continue to accumulate in aging tissues. However, there have been no studies to establish a direct relationship between the accumulation of inflammatory senescent cells and NAD + levels during the aging process. The specific mechanism by which NAD + influences cellular aging remains unclear. Some studies have reported that NAD + levels affect the aging-related secretory phenotype (SASP) of senescent cells [235]. Supplementation with NAD + precursor substances enhances SASP, leading to increased chronic inflammation. CD38 is considered the primary enzyme responsible for NAD + consumption [188, 204], causing NAD + levels to decline during aging. As age increases, CD38 levels also rise, although the underlying mechanism is not well understood.

It has been observed that senescent cells and their SASPs activate CD38 expression in macrophages, promoting CD38-dependent NADase activity [111, 211]. Moreover, CD38 expression is increased in macrophages co-cultured with senescent cells or exposed to conditioned medium [112], suggesting that macrophages may represent the primary cell population responding to SASP with reduced NAD + levels. Another study revealed that cells with dysfunctional mitochondria initiate pro-inflammatory programs by secreting pro-inflammatory cytokines. However, supplementation with NAD + precursors partially ameliorates this situation, in part by reducing inflammation and the burden of senescent cells [127].

## Regulatory mechanism of NAD + metabolism in ovarian aging

### Modifying the properties of enzymes involved in NAD + biosynthesis or degradation

In aging mouse oocytes, the mRNA expression levels of enzymes related to NAD + biosynthesis, such as NAMPT, NaPRT, Nrk1/2, and NMNAT1/3, show no significant changes compared to young oocytes. However, the mRNA and protein expression levels of NMNAT2 significantly decrease [236]. Further investigations revealed that knocking down NMNAT2 in oocytes leads to a reduction in NAD + levels, disrupts the assembly of the meiotic spindle, and perturbs metabolic activities. Rescuing the aging phenotype of oocytes in which NMNAT2 is knocked down through SIRT1 overexpression suggests that NMNAT2 may regulate the oxidative-redox homeostasis of aging oocytes by modulating NAD + levels, thereby suppressing the aging phenotype of oocytes. Additionally, NMNAT2 downregulation can protect cells from p53-dependent cell death in response to DNA damage [237].

Sirtuins, PARPs, and cADPR are the major NAD + -consuming enzymes in cells. Sirtuins have been shown to impact oocyte quality by modulating redox states. In mouse oocytes, the expression of all sirtuins is observed, and their levels gradually decrease until the blastocyst stage. Studies have reported that SIRT1 in GV oocytes can counteract oxidative stress through the Fox3-MnSod axis under in vitro culture conditions [238]. Inhibiting SIRT1 activity in in vitro-cultured oocytes increases the likelihood of spindle and chromosomal abnormalities. SIRT1, FOXO3a, and NRF-1 may form a complex on the SIRT6 promoter, collectively participating in the regulation of ovarian follicle development [239]. P53 protein is expressed in arrested follicles, and SIRT1 can regulate p53 acetylation and p53-dependent apoptosis. SIRT1 activation leads to a reduction in p53 expression, potentially preserving oocytes that would otherwise be lost [240, 241]. Oocyte-specific overexpression of SIRT1 in mice continuously activates FOXO3a and suppresses mTOR, resulting in increased ovarian reserves, extended ovarian lifespan, and enhanced reproductive capacity [242]. Epigenetic inhibitors or RNAi targeting SIRT1 reduce oocyte survival by lowering H4K16ac levels, implying a connection between SIRT1 suppression and the establishment of oocyte follicle development [243]. SIRT3, a mitochondrial sirtuin [244], exhibits reduced expression in aging ovaries, leading to mitochondrial dysfunction and abnormal spindle assembly. Studies have reported that SIRT3 inactivation in in vitro fertilized and cultured embryos increases mitochondrial ROS production, subsequently upregulating p53, resulting in developmental arrest [245]. This indicates a protective role of SIRT3 against oxidative stress-induced developmental arrest in preimplantation embryos cultured in vitro.

**Fig. 3:**
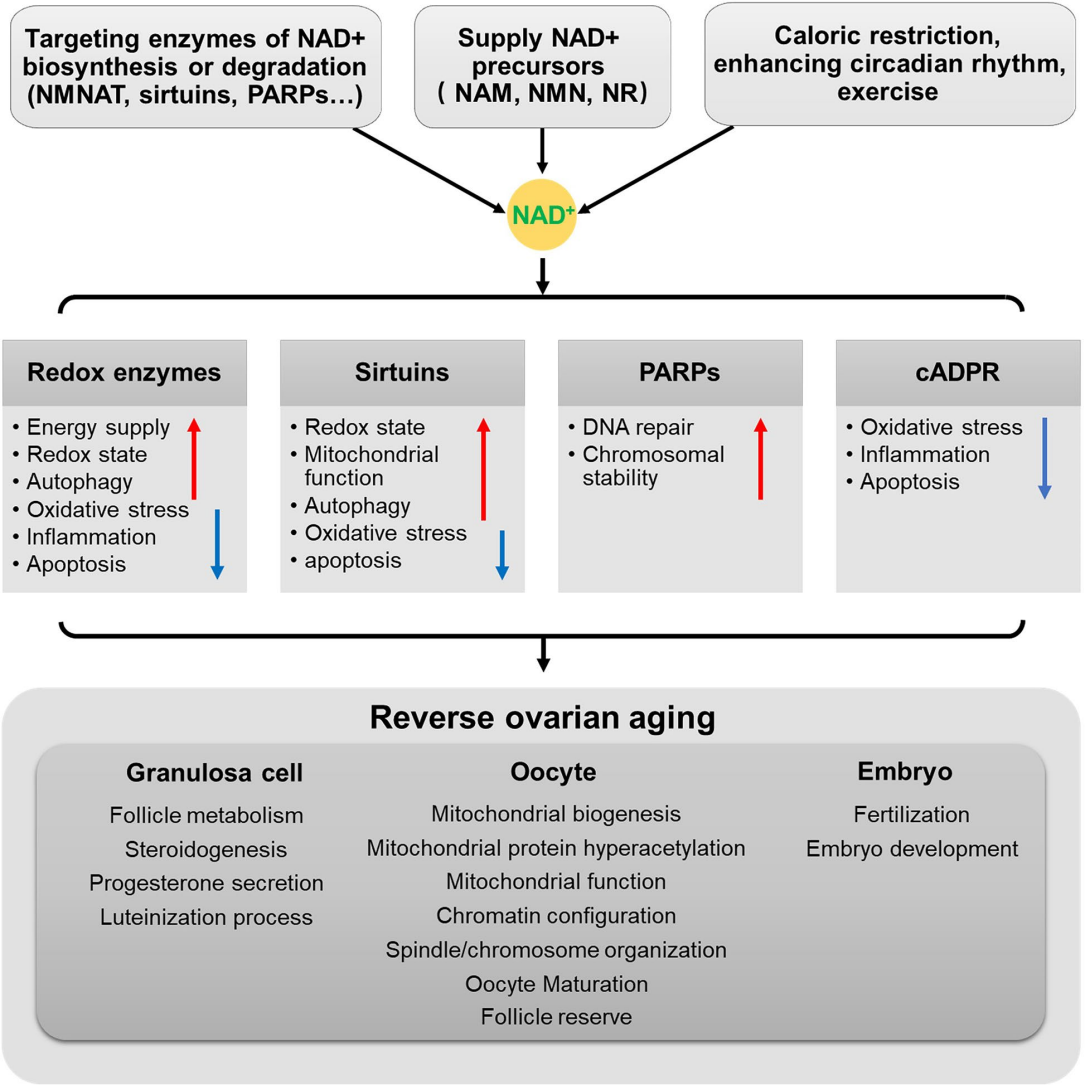
Mechanism of NAD + metabolism in ovarian aging. Modulating the properties of enzymes involved in NAD + biosynthesis or degradation, providing NAD + precursors, and altering lifestyle collectively govern cellular NAD + metabolism. In the context of ovarian aging, NAD + metabolism predominantly exerts a beneficial influence on granulosa cells, oocytes, and embryonic development through alterations in the redox state and the activities of NAD + -dependent enzymes. This ultimately manifests as the reversal of ovarian aging

PARPs maintain chromosome stability during meiosis and play a crucial role in DNA repair [246]. While there is literature suggesting that regulating ovarian NAD + metabolism can reduce DNA damage and maintain genome stability [39, 40, 247], the specific role and mechanisms of PARPs in this context remain unclear. CD38, a representative enzyme for cADPR, can influence cellular calcium signaling, ROS production, and apoptosis. CD38 is not expressed in ovarian follicles but is mainly present in ovarian immune cells, showing an age-dependent increase in expression. CD38 deficiency results in increased NAD + levels in the ovaries, reduced NAM and ADPR levels, and positive regulation of ovarian NAD + metabolism. CD38 deficiency enhances ovarian reserves and reproductive capacity in young female animals, and it can alleviate ovarian inflammation in aging animals by reducing multinucleated macrophage giant cells postreproduction. These beneficial changes are associated with increased ovarian NAD + levels [248] (Fig. 3).

### Supplementing NAD + precursors

In the oocytes of aging mice, protein acetylation levels are abnormally regulated. Sirtuins, a family of NAD + -dependent class III histone deacetylases, also target many nonhistone substrates. NAM is a product of NAD + -dependent enzymes such as sirtuins, PARPs, and CD38, as well as a precursor for NMN and NAD + synthesis. NAM supplementation can increase cellular NAD + levels and noncompetitively inhibit the deacetylase activity of sirtuins. Currently, NAM is known to inhibit the activity of SIRT1 and SIRT2. SIRT1 regulates p53 acetylation and p53-dependent apoptosis in response to DNA damage and oxidative stress [249]. SIRT2 plays a role in microtubule protein deacetylation [250]. Compared to class I and II histone deacetylase inhibitors, NAM can regulate the acetylation and deacetylation of α-tubulin proteins in aging oocytes at lower levels, significantly inhibiting the generation of abnormal microtubule structures during aging and affecting aging-related phenotypes associated with oocyte maturation [251].

Furthermore, NAM treatment significantly reduces the efficiency of GVBD, which produces similar effects to the use of SIRT2-specific inhibitors. However, subsequent events during meiosis I, including spindle assembly and chromosome alignment, remain unaffected. Oocytes treated with NAM show high expression of the anaphase-promoting complex-Cdc20 during meiosis I exit, which is associated with a decrease in cyclin B1 levels and an increase in inhibitory phosphorylation of Cdk1, expected to lead to Cdk1 inactivation and establish a mid-meiotic arrest in meiosis II [252]. Higher concentrations of NMN result in beneficial effects rather than harmful effects in ovulated mouse eggs [251] (Fig. 3).

NMN is a product of the NAMPT reaction and a crucial intermediate in NAD + homeostasis. Supplementing NMN in mice can increase NAD + levels, reverse the aging phenotype of oocytes in aging mice, and mitigate the adverse effects of aging on development when NMN is supplemented in embryo culture medium [8]. However, the exact mechanisms of action remain unclear. SIRT2, through deacetylation and stabilization of BubR1, plays a role in maintaining microtubule-kinetochore attachments, ensuring chromosome separation fidelity. The benefits of NMN for oocytes in aging animals are largely achieved through overexpression of SIRT2. Supplementing NMN can also enhance the fertilization capacity of oocytes by maintaining the dynamics of the cortical granule component ovastacin [39]. Long-term NMN treatment upregulates the expression of PGC-1α, a protein related to mitochondrial function, to a certain extent, reversing damage to granulosa cells in ovarian follicles [253].

Research has shown that NR supplementation can increase NAD + levels in mouse ovarian cells, reversing the ovarian aging phenotype. NR significantly upregulates the expression of genes related to mitochondrial dynamics, such as OPA1, MFN1/2, DRP1, and FIS1, which decrease with ovarian aging. Intermediate metabolites involved in energy metabolism, such as citrate, isocitrate, D-fructose1, and NAD + , are also upregulated, leading to increased ATP production. Mitochondrial biogenesis increases, and the expression of the mitochondrial autophagy-related genes PINK and LC3 is upregulated. NR treatment enhances mitochondrial autophagy and improves mitochondrial dynamics and mitochondrial function in aging oocytes, ultimately improving oocyte quality [40]. Another study reported that NR treatment can mitigate the decline in oocyte quality postovulation and lead to better clinical outcomes in assisted reproductive technology. In this study, the mRNA expression of core proteins of the mitochondrial oxidative phosphorylation chain was studied, and it was found that NR treatment increases the expression of Sdhb, Uqcrc2, and Atp5a1 [247], which helps prevent age-related mitochondrial dysfunction (Fig. 3).

## Therapeutic potential of NAD + in ovarian aging

### Pharmacological NAD + boosting

**Fig. 4:**
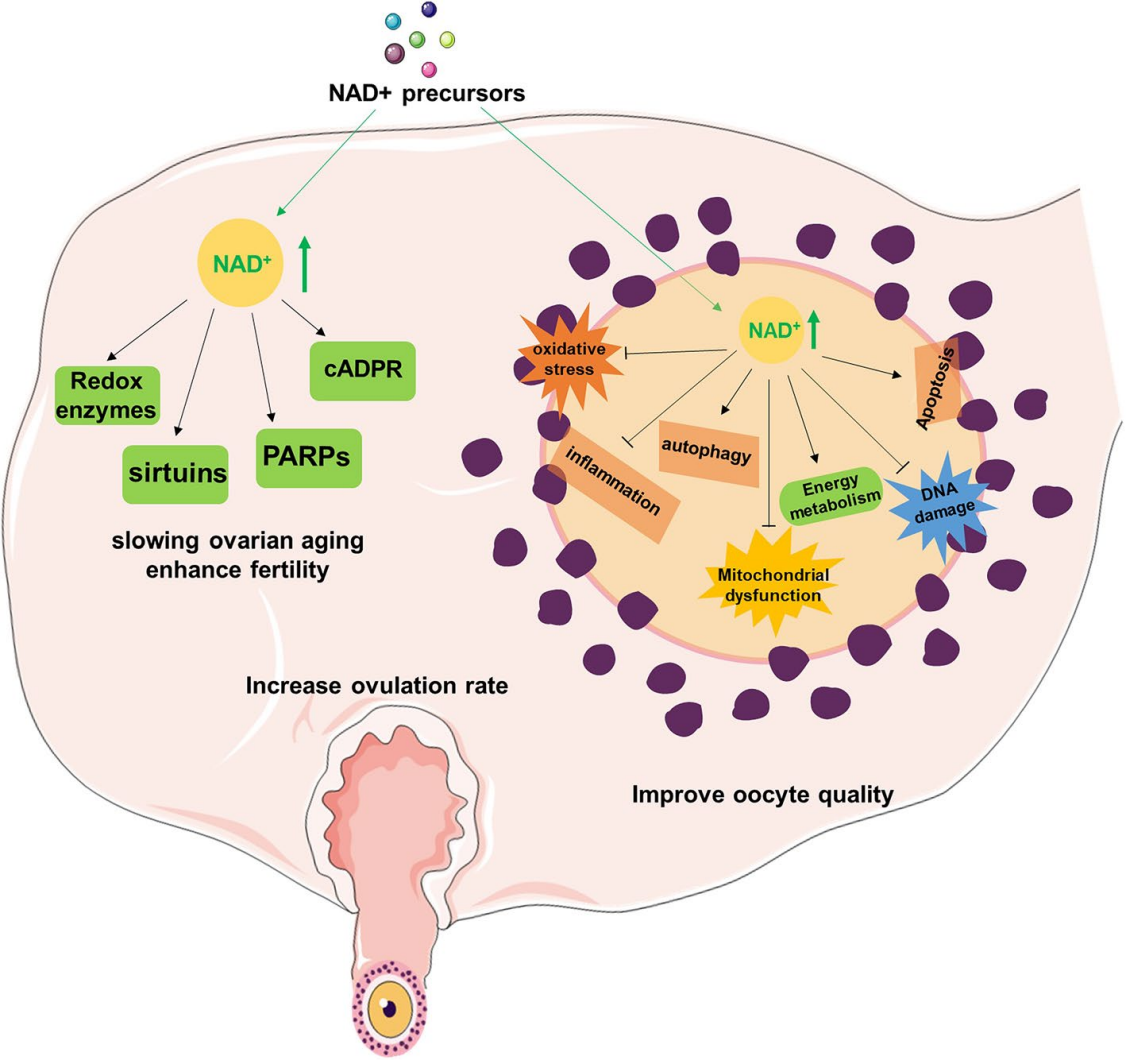
Therapeutic potential of NAD + in ovarian aging. Previous studies conducted in multiple model animals and humans have revealed the feasibility of increasing NAD + levels by supplying NAD + precursors, and the NAD + metabolomic pathway can enhance mitochondrial function, enhance autophagy levels, and maintain protein homeostasis in mitochondria and lysosomes, consequently decelerating the progression of ovarian aging

The levels of NAD + in the body are regulated by a delicate balance between its synthesis and degradation, which is influenced by the aging process [2, 254–257]. The concentration of NAD + in various tissues can be modified through diet, lifestyle, and pharmacological interventions, potentially yielding therapeutic benefits in certain cases. Currently, there are three main strategies for increasing NAD + levels through pharmacology: 1) improving the activity of enzymes involved in NAD + biosynthesis, particularly those that are critical in the rate-limiting steps of both the de novo synthesis and salvage pathways, such as α-amino-β-carboxymuconate-ε-semialdehyde decarboxylase (ACMSD) and NAMPT,2) inhibiting enzymes responsible for NAD + degradation, such as PARPs and CD38; and 3) supplementing the diet with NAD + precursors to support NAD + synthesis through the salvage pathway. Studies conducted in C. elegans [217], flies [258], rodents [259], and humans [260, 261] have revealed the feasibility of increasing NAD + levels by supplying NAD + precursors (Fig. 4).

### Characteristics of NAD + precursors

To date, the identified NAD precursors include tryptophan, NA, NAM, NR, and NMN. These precursors have garnered significant attention as NAD + precursors due to their role in NAD + synthesis, which has been increasingly recognized [260, 262, 263]. Published research has not yet identified any specific preference of ovarian tissue for any of the mentioned NAD precursors. NA and NAM have a long history of being linked to pellagra [264], a disease that is preventable by consuming a diet rich in these precursors [265]. Recently, NR and NMN have gained attention as NAD + precursors due to their comparatively lower side effects compared to NA and NAM. These precursors enter cells through various mechanisms: NA directly passes through cells with the help of membrane carriers (SLC5A8 or SLC22A13) [266, 267], while NR enters cells through equilibrative nucleoside transporters (ENTs). NAM can enter cells either directly or can be converted into NMN via NAMPT. NMN enters cells through specific transporters (the Slc12a8 gene) [268], by conversion into NAM or NR via CD38 or CD73, or through ENTs [159, 188].

In terms of pharmacokinetics, NAD + precursors exhibit distinct properties. Tryptophan, NAM, and NA in plasma present the highest concentrations, surpassing 0.1 μM, with the concentration of NAM being ten times higher than that of NA in plasma. Moreover, under basal conditions, NR and NMN are almost nonexistent in plasma. That is, the liver is the main provider of circulating NAM, accounting for more than 95% of tryptophan-NAM production. The absorption, production, and consumption of NAM and NAD vary significantly among organs, correlating with the expression of enzymes involved in NAD synthesis and consumption. Administration of high doses of NA leads to elevated levels of NAM, which possesses a longer half-life than NA [269]. Although NAM has a stronger ability to raise NAD + levels, excessive NAM concentrations can result in adverse effects, such as nausea and vomiting [270], and may also negatively impact NAD + -consuming enzymes such as PARPs and sirtuins [261]. Clinical studies have shown the effectiveness of NA in raising blood and muscle NAD + levels, thereby alleviating systemic NAD + deficiency in patients with mitochondrial myopathy and improving muscle performance [271].

Likewise, oral intake of NAM can quickly elevate both NAM and NAD + levels in the blood [272]. Research has also shown that NMN can significantly increase NAD + levels in peripheral tissues and can even traverse the blood–brain barrier to increase NAD + in the brain [273, 274]. Upon oral administration, NMN is rapidly absorbed into the bloodstream from the intestine within a short span of 2–3 min, swiftly distributing to diverse tissues through circulation within 15 min. After 15 min, the plasma NMN level returns to baseline, while increased concentrations of NAD + can be detected in the liver, skeletal muscle, and cerebral cortex [275]. Additionally, clinical trials have demonstrated that a single oral dose of NMN can substantially increase the levels of NMN and NAD + metabolites in the plasma [276]. Similarly, oral intake of NR increases NAD + levels in blood cells, displaying 2–3 times greater potency at raising ADPR compared to NAM [261].

### NAD + boosting in vitro

Notably, the potential benefits of supplementation with NAD + precursors in preventing and treating age-related diseases have gained increasing attention. Nevertheless, the impact of NAD + precursor supplementation on ovarian aging remains insufficiently investigated. A few studies have explored the role of NAD + precursors in ovarian aging (Table 1). For instance, a study in 2013 demonstrated that supplying ovulated aging oocytes with NAM could inhibit the formation of abnormal spindles and reduce cell fragmentation [251], implying a potential delay in the aging process. Furthermore, boosting NAD + biosynthesis with NA was found to prevent oxidative stress and meiotic defects in old oocytes, effectively delaying the aging process of oocytes in aged mice [236]. However, conflicting results have been observed in other studies. Specifically, one study found that NAM disrupted the regulation of Cdk1 in ovulated oocytes, impairing entry into meiosis I and the establishment of metaphase II arrest [252]. A recent study revealed the efficacy of NR supplementation in maintaining the quality of postovulatory oocytes [247]. NR effectively curbs the decline in NAD + levels, counteracts mitochondrial dysfunction, preserves spindle and chromosome structure, and decreases ROS levels and DNA damage, thereby improving oocyte quality and embryonic development potential and potentially enhancing the success rate of assisted reproductive technology. The precise molecular mechanism underlying the numerous benefits of NR supplementation remains unclear. Similarly, a separate study found that NMN treatment in embryo culture reverses the negative impact of aging on development [8].

**Table 1:** Therapeutic potential of NAD+ in rodents ovarian aging

Study	Drugs	Concentration	Subjects	Administration pathway	Effects
Lee et al., 2013 [251]	NAM	20 mM	In vitro aging oocytes from 6–8 weeks old mice	Added to medium	cellular fragmentation, spindle elongation and astral microtubules↓
Wu et al., 2019 [236]	NA	50 mM	Oocytes form 42–45 weeks old mice	Added to medium	NAD + content↑ROS levels and the frequency of spindle/chromosome defects↓
Riepsamen et al., 2015 [252]	NAM	10 mM	Oocytes from 7–9 weeks old mice	Added to medium	Disrupted Cdk1 regulation↑ entry into meiosis I and the establishment of metaphase II arrest↓
Li et al., 2023 [247]	NR	0.5 μM	Oocytes or embryos from 6-8 weeks old mice	Added to medium	MII oocytes quality and early embryonic development potential↑normal spindle/chromosome structure↑ mitochondrial dysfunction, ROS levels, DNA damage and apoptosis↓
Bertoldo et al., 2020 [8]	NMN	1 μM	In vivo matured oocytes from 4 weeks or 12 months old mice	Added to medium	Blastocyst formation in embryos derived from aged female oocytes↑ blastocyst cell number in embryos derived from young female oocytes↑
Bertoldo et al., 2020 [8]	NMN	0.5 or 2 g/L	12 to 14 months old mice	Added to drinking water for 4 weeks	NADH levels in oocytes↑spindle assembly, ovulation rate, blastocyst formation rate↑the time to first live birth, the overall proportion of animals achieving live birth↑
Miao et al., 2020 [39]	NMN	200 mg/kg per day	64–68 weeks old mice	Intraperitoneally injection for 10 days	Aged mice oocytes quality, oocytes ovulation rate, fertilization ability↑ meiotic competency, normal spindle/chromosome structure and mitochondrial function↑ROS levels and apoptosis↓
Huang et al., 2022 [253]	NMN	0.5 mg/mL per day	40 weeks old mice	Added to drinking water for 20 weeks	Anti-aging and anti-inflammatory↑estrus cycle condition and endocrine function↑the number of primordial, primary, secondary, antral follicles and corpora luteum of ovary↑mitochondria biogenesis, autophagy level and proteases activity in granulosa cell↑
Yang et al., 2020 [40]	NR	400 mg/kg per day	8 month old mice	Added to drinking water for 4 months	The number of ovarian follicles, ovulatory potential and live birth rate↑ROS levels, spindle anomalies in aging oocytes↓mitochondrial function↑

### NAD + boosting in vivo

The impact of NAD + precursor supplementation on ovarian aging has been explored in vivo (Table 1). The results have shown that NMN supplementation in vivo can enhance the oocyte quality, ovulation rate, and fertility in aged mice [8]. Studies have found that a 4-week preovulation treatment of NMN can restore oocyte spindle assembly and reduce the occurrence of aneuploidy, improving oocyte quality. Additionally, the mechanisms underlying the improvement in the ovulation rate with NMN treatment may be related to the NAD + metabolome and the effects of other tissues on follicle development. Low-dose NMN supplementation has been found to increase the pregnancy rate and the number of live births in aged mice, suggesting an optimal concentration for promoting fertility. Furthermore, NMN treatment can also ensure normal dynamics of cortical granules, improve sperm binding, and enhance the fertilization capacity of aged oocytes [39]. The benefits of NMN treatment are mainly attributed to the enhancement of energy metabolism in oocytes, although a similar effect was observed in aged mice with transgenic overexpression of SIRT2. Nevertheless, the results from SIRT2 knockout mice indicate that SIRT2 protein may not play a crucial role in oocyte function [277, 278]. Consequently, further research is necessary to ascertain the participation of other members of the sirtuin family in mediating the effects of NMN.

A separate study observed similar outcomes when utilizing NMN to boost NAD + biosynthesis in aged mice [39]. The research team discovered that NMN supplementation led to an increase in antral follicles and ovulated oocytes while decreasing the occurrence of mature oocytes with fragmentation. NMN treatment improved the maturation of the nucleus and cytoplasm in aged oocytes, thus boosting the maturation rate of oocytes. Through the implementation of single-cell transcriptome profiling, the researchers investigated the potential effectors of NMN and discovered that the impact of NMN on aged oocytes may be due to its effects on mitochondrial function. Specifically, the benefits of NMN appear to stem from the restoration of mitochondrial function, reduction in the accumulation of ROS, and suppression of apoptosis (Fig. 4). Some studies suggest that administering NMN to 40-week-old mice for 20 weeks can enhance mitochondrial function, enhance autophagy levels, and maintain protein homeostasis in mitochondria and lysosomes, consequently decelerating the progression of ovarian aging [253]. Additionally, prolonged supplementation with NMN was also found to lower the expression of the ovarian aging marker P16 and increase the expression levels of mitochondrial function-related proteins. Furthermore, supplementation with NR was found to restore mitochondrial function and enhance mitochondrial energy metabolism, leading to improved ovarian reserve, increased ovulation potential, and a higher live-birth rate in aged mice [40, 279]. However, currently, there is a lack of available literature that describes clinical studies investigating the use of NAD precursors in research on ovarian aging.

## Therapeutic potential of NAD + in clinical trials on aging-related conditions

Most preclinical rodent studies have revealed the promising translational potential of NAD + boosting therapy. Some clinical trials have already investigated the use of NAD + precursors for aging-related conditions (Table 2). The NAD + precursors used in clinical trials are mainly NR and NMN [276, 280], neither of which has been reported to cause adverse reactions [281], while the use of NA can cause flushing and pain [282]. Clinical trials evaluating the pharmacokinetics and toxicology of NAD + precursors have yielded preliminary evidence supporting the safety of NAD + boosting therapy [283–286]. Nonetheless, translating the promising therapeutic effects observed in preclinical animal models to humans has proven challenging due to the mild and occasionally contradictory nature of the beneficial effects of NAD + precursors.

**Table 2:** Therapeutic potential of NAD + in clinical trials on aging-related conditions

Study	Drugs	Topic	Intervention	Condition
NCT03310034	Trp, NA, NAM	To evaluate the effect of NAD + precursors on mitochondrial function, energy metabolism and physical function	Adults aged 65 to 75 years will take 207.5 mg niacin equivalents or placebo daily for 31 days	Aging Mitochondrial function
NCT03818802	NR	To evaluate the effect of NR in bone, skeletal muscle and metabolic functions in aging	Elderly female volunteers aged 65 to 80 years will take 1000 mg of NR or placebo daily for 6 months	Aging
NCT05483465	NR	To evaluate the effect of NR supplementation on brain vascular health in aging	Adults aged 65 to 80 years in the community will take 1000 mg of NR or placebo daily for 8 weeks	Aging
NCT04907110	NR	To evaluate the effects of exercise training combined with NR supplementation on metabolic health in older individuals	Adults aged 65 to 80 years with a BMI between 25–35 kg/m^2^ will take 1000 mg of NR or placebo daily for 40 days	Overweight and obesity Aging Type 2 diabetes
NCT02921659	NR	To evaluate the efficacy of NR for improving physiological function	For adults aged 55 to 80 years, a placebo crossover will precede a 6-week intervention with 500 mg of NR twice daily	Aging
NCT03821623	NR	To evaluate the efficacy of NR for targeting elevated systolic blood pressure and arterial stiffness	Healthy adults older than 50 years will take 250 or 1000 mg NR or placebo daily for 3 months	Aging
NCT04040959	NR	To evaluate the efficacy of NR for targeting arterial stiffness and elevated systolic blood pressure	Moderate to severe chronic kidney disease patients aged 35 to 80 years will take 500 mg NR or placebo daily for 3 months	Vascular diseases Kidney disease Blood pressure Oxidative stress
NCT04110028	NR	To investigate whether NR can shorten the recovery phase and improve outcome after acute illness	Patients with acute illness over 18 years old will take NR or placebo 50,500,1000 and 2000 mg daily, and each dose lasts for 90 days	Inflammation Acute illness
NCT04220658	/	To evaluate correlations between epigenetic aging and NAD^+^ levels	Biomarkers of aging including NAD^+^ levels in whole blood, interleukins, inflammatory cytokines, growth factors, omega-3 polyunsaturated fatty acids, and patterns in DNA methylation will be measured in adults aged 25 to 80 years old	Epigenetics
NCT05593939	NR	To investigate whether NR can slow aging in human	Healthy adults older than 65 years will be randomized into either an aerobic exercise, time-restricted feeding, NR (2000 mg/day) or control group and followed for 12 weeks	Aging
NCT03501433	NR	To evaluate the effects of NR on metabolism and vascular function	Young (18–35) and older (60–75) adults will take 500 mg NR or placebo daily for 7 days	Aging Lipemia
NCT03432871	NR	To investigate the role of NR in mitochondrial biogenesis	Mitochondrial disease patients aged 18 to 70 years will take 10 mg/kg body weight NR daily for 4 weeks	Mitochondrial diseases Mitochondrial myopathies Mitochondrial DNA deletion
NCT03151239	NMN	To evaluate the effect of NMN on risk factors for diabetes and cardiovascular disease	Postmenopausal and prediabetes women aged 55 to 75 years will take 250 mg NMN or placebo daily for 8	Glucose metabolism disorder
NCT04228640	NMN	To evaluate the efficacy and safety of Uthever (NMN)	Adults aged 45 to 60 years will take 300 mg NMN or placebo daily for 60 days	Aging
NCT04823260	NMN	To evaluate the efficacy and safety of NMN as an anti-aging supplement	Adults aged 40 to 65 years will take 300–900 mg NMN or placebo daily for 60 days	Aging

Positive results have been seen in clinical studies, indicating that NMN supplementation can increase muscle insulin sensitivity in overweight or obese prediabetic women, thereby improving insulin signaling and remodeling [286]. In another clinical investigation of the antiaging effects of NMN, it was observed that NMN can significantly increase NAD levels in the serum of healthy subjects. Furthermore, NMN supplementation leads to increased insulin sensitivity, as evidenced by the substantially reduced HOMA scores compared to those of the control group, highlighting the antiaging effects of NMN [287]. However, a clinical study employing a dose-dependent regimen showed that NMN supplementation did not impact insulin sensitivity, although statistically significant improvements were observed in the health status of the participants [285]. Studies have reported that short-term use of NR has some beneficial effects on healthy older adults [13]. Moreover, long-term supplementation with the NAD + precursor NR exhibits good tolerability and effectively stimulates NAD metabolism in healthy middle-aged and older adults, resulting in reduced levels of circulating inflammatory cytokines [260, 284]. Some studies conducted with older adults have indicated that supplementation with NAD + precursors through L-tryptophan, niacin, and nicotinamide does not improve mitochondrial or skeletal muscle function [283]. Additionally, supplementing NR in older obese men has little effect [280, 288]. Interestingly, a study has reported that NA can rectify systemic NAD deficiency, leading to enhanced muscle performance in patients with mitochondrial myopathy [271].

The reasons behind the absence of beneficial effects in human trials of NAD + boosting therapy thus remain unclear. This could potentially be attributed to the inability of NAD + precursors to increase NAD levels in specific tissues of the human body [260, 263, 289]. Moreover, the duration of the studies may have been inadequate to attain clinical benefits, and the experimental designs primarily concentrated on healthy subjects with normal baseline NAD levels. Therefore, further human clinical trials are imperative to ascertain suitable dosage regimens, treatment periods, and long-term toxicological outcomes. These trials should account for participant diversity to effectively tackle the translational challenges associated with NAD + promotion strategies.

## Perspective

The significance of NAD + in various disease models, including cancer, neurodegeneration, organ disease, and aging, has gained widespread recognition and has received extensive research attention [7, 198, 260, 290, 291]. This is attributed to the essential role of NAD + as a cofactor in redox reactions, as well as its involvement in vital processes such as energy metabolism, cellular homeostasis, posttranslational modification, epigenetic changes, and RNA stability [144, 292–294]. Studies on NAD + boosting by supplementing NAD + precursors in the context of ovarian aging are gaining momentum. Animal models of ovarian aging have demonstrated that supplementation with NAD + precursors improves oocyte quality, alleviates ovarian aging, and enhances fertility.

However, the molecular mechanism behind NAD + precursors remains unclear. Numerous questions have arisen, encompassing the transportation of NAD + and its precursors to ovarian cells and organelles, the preference of ovarian cells for certain NAD + precursors, the effect of specific NAD + metabolic pathways on NAD + flux in healthy and aging ovaries, and the repair mechanism for toxic metabolites produced by NAD + metabolism. Currently, the beneficial effects of NAD + boosting therapy in human trials are highly limited, leaving numerous questions unanswered. Notwithstanding these uncertainties, although much research is required to comprehensively elucidate the biological and therapeutic potential of NAD + in this context, NAD + boosting therapy still holds promise for addressing ovarian aging.

## Data Availability

Not applicable.
